# PReS-FINAL-2269: Incidence of antiphospholipid antibody syndrome in a cohort of children suffering from either arterial or venous thrombosis

**DOI:** 10.1186/1546-0096-11-S2-P259

**Published:** 2013-12-05

**Authors:** P Cancarini, M Cattalini, I Bosio, M Marchini, S Vitari, A Meini

**Affiliations:** 1 Pediatric Immunology and Rheumatology, Pediatric Clinic, University of Brescia, Brescia, Italy

## Introduction

Arterial or venous thrombosis rarely occurs in children, in almost 70% of these patients an acquired or congenital cause can be identified. Hereditary thrombophilia can be identified in 10-30% of children with other evident causes of thrombosis, and in 60% of children with apparent idiopathic thrombosis. Therefore it is suggested that thrombophilia screening should be done in both cases.

## Objectives

Aim of this study was to look for thrombophilia in a cohort of children with thrombotic events.

## Methods

The clinical records of all patients admitted to the Pediatric Clinic, the Pediatric Intensive Care Unit and Neuropsychiatry of the Children Hospital of Brescia between January 2006 and January 2012 were analyzed to select those with either clinical history or discharge diagnosis of an acute thrombotic event. Patients with malignancy were excluded. The selected patients were analyzed for clinical history, characteristics of the thrombotic event and possible predisposing causes and underwent a complete screening for thrombophilia.

## Results

30 out of the 6379 children whose charts had been evaluated met the inclusion criteria; in none of them thrombophilia was previously diagnosed. Mean age was 5.0 ± 3.7 years, 17/30 were males, 19 had arterial and 11 venous thrombosis. Central nervous system was involved in 28 patients, veins of legs in 2. 4 children died during the hospitalization; the other 26 were contacted: 21 agreed to undergo a clinical evaluation and complete screening for thrombophilia.

Family history was significant in 9 patients (4 cerebral ischemia, 1 acute myocardial infarction, 1 cerebral ischemia and myocardial infarction, 1 deep vein thrombosis, 1 LAC positivity and 1 recurrent abortion). Possible predisposing causes were found in 6 children: cardiopathy (2), otomastoiditis (1), trauma (1) venous cannulation (2).

Protein C was low (54% and 61%) in 2 children and Protein S in other 2 (63% and 31%). A child had an heterozygous mutation for Factor II, two (9,5%) hyperomocysteinemia associated with homozygosity for variant C677T of MTHFR. Significant positivity for Antiphospholipid Antibodies was found in 2 children: one for anti-B2GPI IgM (0.504 UO) and one double positivity for anticardiolipin IgG (25.4 GPL) and anti-B2GPI IgG (0.199 UO), allowing the diagnosis of Antiphospholipid Antibody Syndrome (APS).

## Conclusion

Thrombophilia was found in 9 out of the 21 children studied. In 3 children there were both a thrombophilic and acquired predisposing condition: one Protein C deficit and otomastoiditis, one Protein S deficit and cardiopathy and one Antiphospholipid Antibodies Syndrome and cardiopathy. The other 6 children had been found to have hereditary thrombophilia or Antiphospholipid Antibody Syndrome, without any extrinsic predisposing factor and all of them had been previously diagnosed as idiopathic thrombosis. Our study underlines the importance to do a complete screening for thrombophilia in all children with thrombosis. Moreover, even though APS is estimated to be very rare in children, our study underlines that some patients may be misdiagnosed without appropriate screening in case of a child presenting with a thrombotic event.

## Disclosure of interest

None declared.

